# Analysis of the dynamics of a vector-borne infection with the effect of imperfect vaccination from a fractional perspective

**DOI:** 10.1038/s41598-023-41440-7

**Published:** 2023-09-01

**Authors:** Tao-Qian Tang, Rashid Jan, Adil Khurshaid, Zahir Shah, Narcisa Vrinceanu, Mihaela Racheriu

**Affiliations:** 1 Department of Internal Medicine, E-Da Hospital, I-Shou University, Kaohsiung, 82445 Taiwan; 2 School of Medicine, College of Medicine, I-Shou University, Kaohsiung, 82445 Taiwan; 3 International Intercollegiate Ph.D. Program, National Tsing Hua University, Hsinchu, 30013 Taiwan; 4 Department of Family and Community Medicine, E-Da Hospital, I-Shou University, Kaohsiung, 82445 Taiwan; 5 Department of Engineering and System Science, National Tsing Hua University, Hsinchu, 30013 Taiwan; 6 Department of Civil Engineering, Institute of Energy Infrastructure (IEI), College of Engineering, Universiti Tenaga Nasional (UNITEN), Putrajaya Campus, Jalan IKRAM-UNITEN, 43000 Kajang, Selangor Malaysia; 7 Department of Mathematics, University of Swabi, Swabi, 23561 KPK Pakistan; 8 Department of Mathematical Sciences, University of Lakki Marwat, Lakki Marwat, 28420 KPK Pakistan; 9 Faculty of Engineering, Department of Industrial Machines and Equipments, “Lucian Blaga” University of Sibiu, 10 Victoriei Boulevard, Sibiu, Romania; 10 Medicine Faculty, Lucian Blaga University of Sibiu, 2A Lucian Blaga Str, 550169 Sibiu, Romania; 11 Cty Clin Emergency Hosp, 2-4 Corneliu Coposu Str, 550245 Sibiu, Romania

**Keywords:** Computational biology and bioinformatics, Diseases, Mathematics and computing

## Abstract

The burden of vector-borne infections is significant, particularly in low- and middle-income countries where vector populations are high and healthcare infrastructure may be inadequate. Further, studies are required to investigate the key factors of vector-borne infections to provide effective control measure. This study focuses on formulating a mathematical framework to characterize the spread of chikungunya infection in the presence of vaccines and treatments. The research is primarily dedicated to descriptive study and comprehension of dynamic behaviour of chikungunya dynamics. We use Banach’s and Schaefer’s fixed point theorems to investigate the existence and uniqueness of the suggested chikungunya framework resolution. Additionally, we confirm the Ulam–Hyers stability of the chikungunya system. To assess the impact of various parameters on the dynamics of chikungunya, we examine solution pathways using the Laplace-Adomian method of disintegration. Specifically, to visualise the impacts of fractional order, vaccination, bite rate and treatment computer algorithms are employed on the infection level of chikungunya. Our research identified the framework’s essential input settings for managing chikungunya infection. Notably, the intensity of chikungunya infection can be reduced by lowering mosquito bite rates in the affected area. On the other hand, vaccination, memory index or fractional order, and treatment could be used as efficient controlling variables.

## Introduction

The study of vector-borne illnesses has gotten a lot of interest recently, and mathematics has shown to be a beneficial tool in these investigations. For illnesses such as dengue fever, malaria, chikungunya, and the human immunodeficiency virus, several deterministic models with a temporal dimension have been developed to study chikungunya infection. Chikungunya is a viral infection caused by the chikungunya virus, which belongs to the *Alphavirus* genus within the Togaviridae family. The disease is transmitted to individuals through the bites of $\textit{aedes aegypti}$ mosquitoes^1^. The first outbreak of chikungunya, which presented symptoms similar to dengue fever, was reported in 1952 on the Comoros Islands, positioned off the shoreline of northern Mozambique^2^. During its inception, this viral infection has caused epidemics in Asian, African, European, and American nations at unpredictable intervals of 2–20 years. Chikungunya infection was first reported in Bangkok, Thailand in 1958^3^, followed by its emergence in India during the early 1960s in places like Vellore, Calcutta, and Maharashtra^4^. Subsequently, it spread across Sri Lanka and other Southeast Asian nations including Myanmar, Indonesia, and Vietnam in 1969^4^. Thailand saw sporadic cases of chikungunya fever between 1976 and 1995, and the virus resurfaced in the Democratic Republic of Congo in 1999-2000^5^. In 2004, 13,500 cases were recorded in Lamu, Kenya^5^. The epidemic hit the Reunion Islands of the Indian Ocean from 2005 to 2007, and 197 cases were reported in Europe in 2007^1^. During the epidemic, the virus mutated, making it more easily transmissible by the tiger mosquito $\textit{aedes albopictus}$. As per research, areas with the presence of tiger mosquitoes may have a greater likelihood of a chikungunya outbreak.

The incubation time of this viral infection is typically 3–7 days following a harmful sting from a vector afflicted with an infectious virus, with fever being the most common symptom. Chikungunya fever symptoms are distinct from those of a typical fever in that they are accompanied by severe joint discomfort. Nausea, rashes, headaches, and exhaustion are other frequent complaints. Some instances may also result in neurological, retinal, or carpological issues, making recovery more harder for elderly people than for younger people. People might suffer from joint discomfort for years at a time, implying that the healing process can take a lengthy time. Chikungunya symptoms are often minor, although the disease is commonly mistaken as zika or dengue due to symptom overlap. Just a few instances of chikungunya have resulted in death, and the majority of infected persons will recover entirely and obtain lasting immunity. As a result, there is no vaccination or treatment for chikungunya. Only using drugs for short comfort can help control the symptoms. Mosquito breeding areas should be examined to avoid the spread of illness. One can prevent insect bites by utilizing mosquito repellents and opting to wear clothing with long sleeves and full-length coverage^1^.

The rapid proliferation of mosquito breeding grounds has been associated with the increasing interconnectedness of global populations and the ongoing effects of climate change. This pressing concern necessitates the advancement of vector control strategies and the establishment of monitoring indices to effectively manage such programs. Mathematical modeling has emerged as a valuable tool to analyze epidemic diseases, particularly during the twentieth century, and has facilitated the development of optimal control techniques for various infectious diseases. In the context of chikungunya virus infection, Dumont et al. proposed a model that relied on the temporal data from the initial chikungunya epidemic in several municipalities on Reunion Island^8^. Moulay et al. presented a model that depicts the intricate dynamics of mosquito populations and their viral transmission to humans^9^. Yakob & Clements^10^ develop a model that provided a close estimation of the outbreak’s peak incidence and ultimate epidemic size. This model was further explored by Naowarat and Tang who incorporated the existence of two $\textit{Aedes}$ mosquito species^11^. The information about chikungunya by WHO^1^ employed three scenarios to estimate disease control measures for chikungunya virus, which encompassed situations involving a single vector, two vectors, and two vectors with human and non-human reservoirs. They emphasized the significance of regularly assessing the effectiveness of insect prevention techniques. Agusto and his colleagues developed a chikungunya model that considered three age-structured transmission phenomena, examining the transition dynamics of individuals among different stages, including juvenile, adult, and senior populations^13^. These efforts contribute to the overall understanding of chikungunya virus transmission and aid in devising effective control strategies to combat its spread.

Fractional calculus has emerged as a powerful tool for analyzing and modeling complex systems, including infectious disease dynamics^14,15^. In the context of epidemic models, fractional-order differential equations offer a more accurate and versatile approach to describing the intricate dynamics observed in real-world outbreaks^16,17^. Jan et al.^18^ investigated the transmission dynamics of rift valley fever with vaccination policy in fractional framework. They proved that the results of fractional derivative are more accurate and flexible than the classical derivative. Jan et al. also presented an epidemic model for HIV^19^ through fractional derivative with real data. The authors showed that the fractional models provide more accurate results due to an extra parameter in the system. A fractional order epidemic model has been introduced for pneumococcal pneumonia infection to understand the spread of the infection^20^. As vector-borne disease exhibits knowledge of its previous stages through an associative learning mechanism. The memory within the host population, linked to individual awareness, leads to a decreased rate of contact between vectors and hosts. On the other hand, mosquitoes utilize their past experiences regarding human location, blood preference, color, and human defensive behaviors to select a suitable human host for feeding^21,22^. The incorporation of fractional-order systems in mathematical modeling of vector-borne infection can effectively capture and represent these types of phenomena. In this study, our main goal is to explore the transmission dynamics of chikungunya virus infection while considering treatment, vaccination, and memory index factors. To achieve this, we employ fractional differential equations, which are commonly used in modeling such infectious diseases.

The following is how the manuscript is structured: Section "Theory of fractional calculus" introduces the essential ideas and results of fractional calculus. Section "Evaluation of the dynamics" presents a compartmental model that includes vaccination, treatment, and a memory index to create a more accurate model understanding of the transmission mechanisms of chikungunya. In Sect. "Theory of existence", the proposed model is examined and analyzed. Section "Ulam–Hyers stability" establishes the appropriate conditions for the Ulam–Hyers stability of the model. Section "Solution of our fractional system" discusses a numerical method in order to solve the recommended fractional framework and provides a numerical analysis of the chikungunya dynamics with respect to various parameters. Finally, the article concludes with a summary and final remarks in Sect. "Concluding Remarks".

## Theory of fractional calculus

This section will go through the basic definitions and concepts of fractional calculus, which will be used to investigate our system. The ability to integrate memory effects, which are critical for comprehending vector-borne virus transmission. Furthermore, fractional calculus has many applications in diverse fields of study. We have covered the basics of the Caputo fractional operator, which we will utilise to analyse our proposed model.

### Definition 2.1

 If we examine a function *g*(*k*) in the Lebesgue integrable space $L^{1}([a, b], {\mathbb {R}})$^23^, the fractional integration can be characterised as follows

1 $$\begin{aligned} I_{a^{+}}^{a}g(k)=\frac{1}{\Gamma (\vartheta )}\int _{0}^{k}(k-L)^{\vartheta -1}g(L)dL, \end{aligned}$$

where $\vartheta$ is the fractional order in such a way that $0< \vartheta \le 1$.

### Definition 2.2

 Consider a function *g*(*k*) such that $g(k) \in B^{n}[a, b]$^23^, and the fractional derivative in Caputo form is provided by

2 $$\begin{aligned} ^{b}D_{0^{+}}^{\vartheta }V(k)=\frac{1}{\Gamma (n-\vartheta )}\int _{0}^{k}(k-L)^{n-\vartheta -1}g^{n}(L)dL; \end{aligned}$$

with the condition that $0 < \vartheta \le 1$.

### Lemma 2.1

 Assuming the fractional system stated as follows^23^,

3 $$\begin{aligned} \left\{ \begin{array}{rcl} ^{b}D_{0^{+}}^{\vartheta }{\mathfrak {c}}(k)=&{} C(k), k\in [0,\upsilon ],n-1< \vartheta < n,\\ {\mathfrak {c}}(0)=C_{0} \end{array}\right. \end{aligned}$$

where $C(k) \in B([0,\upsilon ])$, the response of the aforementioned fractional framework is then

4 $$\begin{aligned} {\mathfrak {c}}(k)=\sum _{l=0}^{n-1}d_{l}k^{l}, \ where \ d_{l} \in R, \ \ l=0, 1, \dots ,n-1. \end{aligned}$$

### Definition 2.3

 The Laplace transformation is presented for the Caputo Fractional operator in the following way^24^,

5 $$\begin{aligned} \pounds [^{b}D_{0^{+}}^{\vartheta }{\mathfrak {c}}(k)]=L^{\vartheta }g(L)-\sum _{l=0}^{n-1}L^{\vartheta -l-1}{\mathfrak {c}}^{l}(0), n-1< \vartheta < n. \end{aligned}$$

In addition, consider

6 $$\begin{aligned} ||{\mathfrak {c}}||=\max _{k \in [0,\upsilon ]}\{|{\mathfrak {c}}|, for~~all~~{\mathfrak {c}} \in Z \}, \end{aligned}$$

be the standard deviation specified on $Z = B([0, \upsilon ])$ in which *Z* is a Banach’s space.

### Theorem 2.1

 Consider a Banach space *Z* in which $F:Z \rightarrow Z$ is smooth and confined^25^. If the following set

7 $$\begin{aligned} E=\{{\mathfrak {c}} \in Z: {\mathfrak {c}}=\lambda F{\mathfrak {c}}, \lambda \in (0,1)\} \end{aligned}$$

is limited, then *F* is a fixed point.

## Evaluation of the dynamics

We offer a computational framework that represents the propagation of chikungunya virus infection in this portion of the research. The entire population of both hosts and vectors at a particular moment is represented by $$N_h(t)$$ and $$N_v(t)$$, accordingly. The vector community $$N_v(t)$$ is split into two categories: the susceptible class $$S_v(t)$$ and the infected class $$I_v(t)$$. Similarly, the host community is separated into four groups: the susceptible $$S_h(t)$$, the vaccinated $$V_h(t)$$, the infected $$I_h(t)$$, and the recovered $$R_h(t)$$. The vector community $$N_v(t)$$ is a combination of the $$S_v(t)$$ and $$ I_v (t)$$ classes, whereas the total host population is the sum of $$S_h(t)$$, $$V_h(t)$$, $$I_h(t)$$ and $$R_h(t)$$. In this formulation, $$\mu _h$$ and $$\mu _v$$ represents the natural mortality rates for both kinds of individuals, accordingly. A fraction *p* of susceptible individuals are vaccinated and a fraction $$\tau $$ of the infected class move to the recover class after successful treatment. We represent the dynamics chikungunya infection with the effect of vaccination and vertical transmission is given by

8 $$\begin{aligned} \left\{ \begin{array}{rcl} \frac{dS_h}{dt} &{}=&{} \mu _h N_h -\frac{\beta _1 b S_hI_v}{N_h}-pS_h-\mu _h S_h, \\ \frac{dV_h}{dt} &{}=&{} pS_h-\frac{\beta _2 b V_h I_v}{N_h}-\mu _h V_h,\\ \frac{dI_h}{dt} &{}=&{} \frac{\beta _1 b S_h I_v}{N_h}+ \frac{\beta _2 b V_h I_v}{N_h}-(\tau +\mu _h+\gamma ) I_h, \\ \frac{dR_h}{dt} &{}=&{} \tau I_h+\gamma I_h-\mu _h R_h, \\ \frac{dS_v}{dt} &{}=&{} \mu _v N_v-\frac{\beta _3 b S_v I_h}{N_h}-\mu _v S_v, \\ \frac{dI_v}{dt} &{}=&{} \frac{\beta _3 b S_v I_h}{N_h}-\mu _2 I_v, \end{array}\right. \end{aligned}$$

with

$$\begin{aligned} \mathrm {S_h(0)\ge 0, V_h(0)\ge 0, I_h(0)\ge 0, R_h(0)\ge 0}, \end{aligned}$$

and

$$\begin{aligned} \mathrm {S_v(0)\ge 0, I_v(0)\ge 0.} \end{aligned}$$

In our formulation, the rate of recruitment of humans is represented by $\mu _h N_h$ where $\mu _h$ represent the nativity and mortality rates of humans. In vector inhabitants, the infection outlay from susceptible class to infected class is given by $\beta _3$. On the other hand, the host population is recruited by a rate $\mu _v N_v$ where $\mu _v$ is the birth and death rate of vectors. The transmission rates from susceptible host to infected is indicated by $\beta _1$ and $\beta _2$. One of the latest and most newly prominent disciplines of mathematics is fractional calculus, which works with derivatives and integrals of real and complex classes. In truth, although being as old as the original calculus, this type of calculus has piqued the interest of scholars from a variety of disciplines due to the astonishing results obtained when some of these scholars employed fractional operations to describe real-world difficulties. To better understand the spreading phenomenon more properly, we characterize the dynamics of chikungunya infection using a Caputo-derivative in the following manner:

9 $$\begin{aligned} \left\{ \begin{array}{ll} ^{B}_0 D^{\xi }_k S_h = \mu _h^{\xi } N_h -\frac{\beta _1 b^{\xi } S_hI_v}{N_h}-p^{\xi }S_h-\mu _h^{\xi } S_h, \\ ^{B}_0 D^{\xi }_k V_h = p^{\xi }S_h-\frac{\beta _2 b^{\xi } V_h I_v}{N_h}-\mu _h^{\xi } V_h, \\ ^{B}_0 D^{\xi }_k I_h = \frac{\beta _1 b^{\xi } S_h I_v}{N_h}+ \frac{\beta _2 b^{\xi } V_h I_v}{N_h}-(\tau ^{\xi }+\mu _h^{\xi }+\gamma ^{\xi }) I_h, \\ ^{B}_0 D^{\xi }_k R_h = \tau ^{\xi } I_h+\gamma ^{\xi } I_h-\mu _h^{\xi } R_h, \\ ^{B}_0 D^{\xi }_k S_v = \mu _v^{\xi } N_v-\frac{\beta _3 b^{\xi } S_v I_h}{N_h}-\mu _v^{\xi } S_v, \\ ^{B}_0 D^{\xi }_k I_v = \frac{\beta _3 b^{\xi } S_v I_h}{N_h}-\mu _v^{\xi } I_v. \end{array} \right. \end{aligned}$$

where $^{B}_0 D_k ^{\xi }$ is the derivative of Liouville-Caputo. $\xi$ represents the storage index in this approach. ${\mathcal {E}}_0$ and is given by ${\mathcal {E}}_0(S_{h}^0,V_{h}^0,I_h^0,R_h^0,S_v^0,I_v^0)=(\frac{\mu _h^\xi N_h^0}{p^\xi +\mu _h^\xi }, \frac{p^\xi S_h^0}{\mu _h^\xi },0,0,N_v^0,0 )$ denotes the disease-free steady-state of our suggested fractional framework (9) of chikungunya illness. The aforementioned framework of chikungunya illness threshold parameter is offered by

$$\begin{aligned} {\mathcal {R}}_0=\sqrt{\frac{\beta _3 b^\xi S_v^0}{N_h^0 N_h^0 } \bigg [ \frac{\beta _1 b^\xi S_h^0 +\beta _2 b^\xi V_h^0}{\mu _v^\xi (\tau ^\xi +\mu _h^\xi +\gamma ^\xi )} \bigg ]}. \end{aligned}$$

For our system of chikungunya infection, the existence and uniqueness of the solution will be investigated in the upcoming result. The below result can easily be determined through analytic skills.

### Theorem 3.1

The solutions of the recommended system (9) of chikungunya virus infection are positive and bounded for positive initial condition of state-variable.

### Sensitivity analysis

Sensitivity analysis is a critical tool used in various scientific disciplines, including epidemiology, to assess the sensitivity of different interventions of a biological system. In the context of epidemic modeling, sensitivity analysis helps understand how uncertainties or variations in the model’s parameters impact the predictions and conclusions drawn from the model. Local sensitivity analysis and global sensitivity analysis are two different approaches used in sensitivity analysis to evaluate the impact of input parameter variations on the output of an intervention^26^. In local sensitivity analysis, one parameter is varied at a time, while all others are kept fixed at their baseline values. This allows researchers to observe the individual impact of each parameter on the system output. Global sensitivity analysis considers the interactions between multiple parameters simultaneously. It assesses the collective influence of all parameters on the system’s output and provides a more comprehensive understanding of their combined effects^27^.

**Table 1:** PRCC and *p* values of significant test for ${\mathcal {R}}_0$.

Parameter	Interpretation	PRCC values	*p* values
$\beta _3$	Transmission rate from $S_v$ to $I_v$	0.8448	0.0000
*b*	Biting rate of mosquitoes	0.4447	0.0000
$\xi$	Fractional order or index of memory	0.5807	0.0000
$\beta _{1}$	Transmission rate from $S_h$ to $I_h$	0.6638	0.0000
$\mu _h$	Natural death rate of hosts	–0.2531	0.0000
*p*	Vaccination fraction of susceptible	0.0602	0.0512
$\beta _{2}$	Transmission from $V_h$ to $I_h$	0.5900	0.0000
$\tau$	Recovery due to treatment	–0.2770	0.0000
$\gamma$	Recovery rate from infected hosts	–0.2376	0.0000
$\mu _v$	Natural death rate of vectors	–0.6858	0.0000

**Figure 1:**
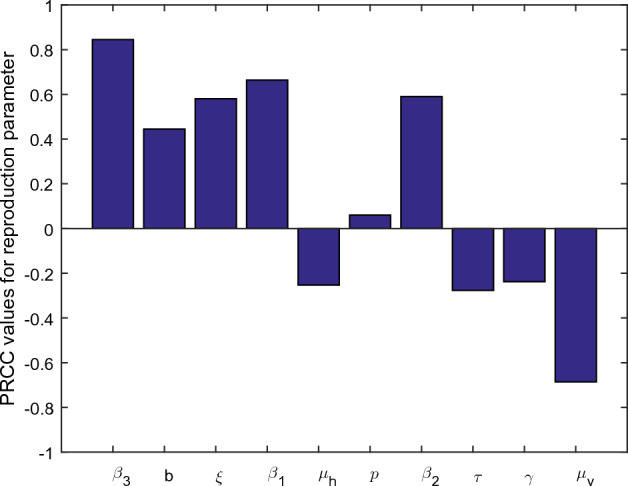
Graphical view of the PRCC sensitivity test for ${\mathcal {R}}_0.$.

Global sensitivity analysis provides a more comprehensive assessment of parameter influences, especially when dealing with complex and nonlinear models or when interactions between parameters are essential for accurate predictions. Here, we will perform global sensitivity analysis of the basic reproduction number of our system. We will use the well-known partial rank correlation coefficient method (PRCC) to show the impact of input parameters on the output of ${\mathcal {R}}_0$. The significant test provides PRCC and p-values for each parameter, indicating that the parameter with the highest PRCC and lowest p-values is the most sensitive parameter in the system. From Fig. 1 and Table 1, we noticed that the input parameters $\beta _3$, $\mu _v$, $\beta _1$, $\beta _2$ and $\xi$ are the most sensitive parameters in the basic reproduction number with PRCC 0.8448, $-0.6858$, 0.6638, 0.5900 and 0.5807, respectively. Therefore, these parameters are recommended to the policy makers and health officials for the prevention and control of the infection.

## Theory of existence

The suggested fractional algorithm’s fundamental framework (9) will be described below. This is accomplished in the subsequent style:

10 $$\begin{aligned} \left\{ \begin{array}{rcl} W _{1}(k, S _{h},V _{h},I _h,R _h,S _v,I _v)&{}=&{} \mu _h^{\xi } N_h -\frac{\beta _1 b^{\xi } S_hI_v}{N_h}-p^{\xi }S_h-\mu _h^{\xi } S_h, \\ W _{2}(k, S _{h},V _{h},I _h,R _h,S _v,I _v)&{}=&{} p^{\xi }S_h-\frac{\beta _2 b^{\xi } V_h I_v}{N_h}-\mu _h^{\xi } V_h, \\ W _{3}(k, S _{h},V _{h},I _h,R _h,S _v,I _v)&{}=&{} \frac{\beta _1 b^{\xi } S_h I_v}{N_h}+ \frac{\beta _2 b^{\xi } V_h I_v}{N_h}-(\tau ^{\xi }+\mu _h^{\xi }+\gamma ^{\xi }) I_h, \\ W _{4}(k, S _{h},V _{h},I _h,R _h,S _v,I _v)&{}=&{} \tau ^{\xi } I_h+\gamma ^{\xi } I_h-\mu _h^{\xi } R_h, \\ W _{5}(k, S _{h},V _{h},I _h,R _h,S _v,I _v)&{}=&{} \mu _v^{\xi } N_v-\frac{\beta _3 b^{\xi } S_v I_h}{N_h}-\mu _v^{\xi } S_v, \\ W _{6}(k, S _{h},V _{h},I _h,R _h,S _v,I _v)&{}=&{} \frac{\beta _3 b^{\xi } S_v I_h}{N_h}-\mu _v^{\xi } I_v,\\ \end{array}\right. \end{aligned}$$

The aforementioned chikungunya viral infection model (10) could be represented in the following manner

11 $$\begin{aligned} \left\{ \begin{array}{rcl} ^{b}D_{0^{+}}^{\vartheta }{} W (k)=&{} Q (k,W (k)),~ k\in [0,\upsilon ],~ 0< \vartheta \le 1,\\ W (0)=W _{0}, \end{array}\right. \end{aligned}$$

where

12 $$\begin{aligned} \left\{ \begin{array}{rcl} W (k)=&{}S_{h}(k),\\ {} &{}V_{h}(k),\\ {} &{} I_h(k),\\ {} &{}R_h(k),\\ {} &{}S_{v}(k),\\ {} &{}I_v(k).\end{array}\right. \left\{ \begin{array}{rcl} W _{0}(k)=&{}S_{h0},\\ {} &{}V_{h0},\\ {} &{}I_{h0},\\ {} &{}R_{h0},\\ {} &{}S_{v0},\\ {} &{}I_{v0}. \end{array}\right. \left\{ \begin{array}{rcl} Q (k,W (k))=W _{1}(k, S_{h},V_{h}, I_h, R_h, S_{v}, I_v) \\ W _{2}(k, S_{h},V_{h}, I_h, R_h, S_{v},I_v)\\ W _{3}(k, S_{h},V_{h}, I_h, R_h, S_{v},I_v)\\ W _{4}(k, S_{h},V_{h}, I_h, R_h, S_{v},I_v)\\ W _{5}(k, S_{h},V_{h}, I_h, R_h, S_{v},I_v)\\ W _{6}(k, S_{h},V_{h}, I_h, R_h, S_{v},I_v) \end{array}\right. \end{aligned}$$

Using Lemma (2.1), the corresponding integral form of the previous (11) is

13 $$\begin{aligned} W (k)=W _{0}(k)+\frac{1}{\Gamma (\alpha )}\int _{0}^{k}(k-s)^{\alpha -1}{} Q (s,W (s))ds, \end{aligned}$$

The following parameters are the major phases in analysing our suggested system:

*(A1)* The variables $K _{Q }, M _{Q }$, and $q \in [0, 1)$ could be found in the following way:

14 $$\begin{aligned} |Q (k,W (k))|\le K _{Q }|W |^{q}+M _{Q }. \end{aligned}$$

*(A2)* The variables $$L Q > 0$$, and all $$W $$, $$\bar{W }\in Z$$ could be found in the following way:

15 $$\begin{aligned} |Q (k,W )-Q (k,\bar{W })|\le L_{Q }[|W -\bar{W }|]. \end{aligned}$$

In this portion, we define a map *F* on *Z* as follows

16 $$\begin{aligned} FW (k)=W _{0}(k)+\frac{1}{\Gamma (\alpha )}\int _{0}^{k}(k-s)^{\alpha -1}{} Q (s,W (s))ds. \end{aligned}$$

If assumptions *(A1)* and *(A2)* are valid, then the framework (11) has a minimum of a single answer. This concept will be used to our suggested approach to chikungunya virus transmission.

### Theorem 4.1

If assumptions $\textbf{A1}$ and $\textbf{A2}$ are valid, the suggested fractional model (9) of chikungunya viral infection has a minimum of a single solution.

Proof 4.1. We shall utilise Schaefer’s fixed point theorem to demonstrate the intended outcome. This theorem will be illustrated over four stages, outlined below:

**S1:** In this stage, we shall demonstrate the consistency of *F*. In this case, we presume that $W _{i}$ is continuous for $i = 1, 2, \dots , 9$, which means that $Q (k, W (k))$ is also continual. We select $W _{j}$, $W \in Z$ in such a way where $W _{j} \rightarrow W$, and $FW j \rightarrow FW$ is required. Next, we’ll look at:

17 $$\begin{aligned} ||FW _{j}-FW ||= & {} \max _{k \in [0,\upsilon ]}\bigg |\frac{1}{\Gamma (\alpha )}\int _{0}^{k}(k-s)^{\alpha -1}Q_{j}(s,W _{j}(s))ds- \frac{1}{\Gamma (\alpha )}\int _{0}^{k}(k-s)^{\alpha -1}{} Q (s,W (s))ds\bigg | \\\\\le & {} \max _{k \in [0,\upsilon ]}\int _{0}^{k}\bigg |\frac{(k-s)^{\alpha -1}}{\Gamma (\alpha )}\bigg ||Q _{j}(s,W _{j}(s))-Q (s,W (s))|ds\\\\\le & {} \frac{\upsilon ^{\alpha }L_{Q }}{\Gamma (\alpha +1)}||W _{j}-W ||\rightarrow 0 \ \ as \ \ j\rightarrow \infty . \end{aligned}$$

Insofar as $$Q $$ is persistent, $$FW j \rightarrow FW $$; as a consequence, the operator *F* is also continual.

**S2:** The second stage tends to determine the boundedness of the *F* expression. If $W \in Z$, then the operator *F* must satisfy the subsequent scenario

18 $$\begin{aligned} ||FW ||= & {} \max _{k \in [0,\upsilon ]}\bigg |W _{o}(k)+\frac{1}{\Gamma (\alpha )}\int _{0}^{k}(k-s)^{\alpha -1}{} Q (s,W (s))ds\bigg |\nonumber \\\le & {} |W _{0}|\max _{k \in [0,\upsilon ]}\frac{1}{\Gamma (\alpha )}\int _{0}^{k}|(k-s)^{\alpha -1}||Q (s,W (s))|ds\nonumber \\\le & {} |W _{0}|+\frac{\upsilon ^{\alpha }}{\Gamma (\alpha +1)}[K_{Q}||W ||^{q}+M_{Q }]. \end{aligned}$$

Our goal is therefore to demonstrate the boundedness of $$F(S )$$ for a bounded subgroup $$S $$ of Z. Consider arbitrary $$W \in S$$; because the set *S* is limited, we may discover a $$K\ge 0$$ in the following way

19 $$\begin{aligned} ||W ||\le K, \forall W \in S. \end{aligned}$$

As a result of extending the preceding criterion to any $W \in S$, we get

20 $$\begin{aligned} ||F{\mathcal {W}}||\le |W _{0}|+\frac{\upsilon ^{\alpha }}{\Gamma (\alpha +1)}[K_{Q }||W ||^{q}+M_{Q }]\le |W _{0}|+ \frac{\upsilon ^{\alpha }}{\Gamma (\alpha +1)}[K_{Q }K^{q}+M_{Q }]. \end{aligned}$$

Hence, *F*(*S*) is bounded.

**S3:** In the third stage, we suppose $k_{1}, k_{2} \in [0, \upsilon ]$ in such a way that $k_{1}\ge k_{2}$ to demonstrate the equi-continuity, and then

21 $$\begin{aligned} |FW (k_{1})-FW (k_{1})|= & {} \bigg |\frac{1}{\Gamma (\alpha )}\int _{0}^{k_{1}}|(k_{1}-s)^{\alpha -1}||Q (s,W (s))|ds-\frac{1}{\Gamma (\alpha )}\int _{0}^{k_{2}}|(k_{2}-s)^{\alpha -1}||Q (s,W (s))|ds\bigg |\nonumber \\\le & {} \bigg |\frac{1}{\Gamma (\alpha )}\int _{0}^{k_{1}}|(k_{1}-s)^{\alpha -1}|-\frac{1}{\Gamma (\alpha )}\int _{0}^{k_{2}}|(k_{2}-s)^{\alpha -1}|\bigg ||Q (s,W (s))|ds\nonumber \\\le & {} \frac{\upsilon ^{\alpha }}{\Gamma (\alpha +1)}[K_{Q }||W ||^{q}+M_{Q }][k_{1}^{\alpha }-k_{2}^{\alpha }]\rightarrow 0 ~as ~k_{1}\rightarrow k_{2}. \end{aligned}$$

As a result, Arzela-Ascoli’s theorem suggests that *F*(*S*) is relatively concise.

**Step 4**: In the final phase, we select the subsequent set up

22 $$\begin{aligned} E =\{W \in Z: W =\lambda FW , \lambda \in (0,1)\}. \end{aligned}$$

To demonstrate the boundedness of the group $$E $$, let $$W \in E $$, than for any $$k \in [0, \upsilon ]$$, the argument that follows is true

23 $$\begin{aligned} ||W ||=\lambda ||FW ||\le \lambda \bigg [|W _{0}|\frac{\upsilon ^{\alpha }}{\Gamma (\alpha +1)}[K_{Q }||W ||^{q}+M_{Q }\bigg ]. \end{aligned}$$

This indicates that the group $$E $$ is finite. As a consequence of Schaefer’s theorem, the variable *F* has a definite position; as a result, it provides a minimum of one remedy to our suggested scheme (11).

### Remark 4.1

If the requirement $$(H1 $$) for $$q = 1$$ is fulfilled, the conclusion of Theorem (4.1) is satisfied for $$\frac{\upsilon ^{\alpha } {\mathcal {K}}_{{\mathcal {Q}}}}{\Gamma (\alpha +1)}<1$$.

### Theorem 4.2

If the premise $\frac{\upsilon ^{\alpha } {\mathcal {K}}_{{\mathcal {Q}}}}{\Gamma (\alpha +1)}<1$ is valid, then our desired fractional framework (11) has a distinctive answer.

Proof 4.2. For the desired outcome, assuming $W ,\bar{W } \in Z$ and using Banach’s contraction theorem, we get

24 $$\begin{aligned} ||FW -F\bar{W }||\le & {} \max _{k \in [0,\upsilon ]}\frac{1}{\Gamma (\alpha )}\int _{0}^{k}|(k-s)^{\alpha -1}||Q (s,W (s))-Q (s,\bar{W }(s))|ds\nonumber \\\le & {} \frac{\upsilon ^{\alpha } {\mathcal {K}}_{Q }}{\Gamma (\alpha +1)}||W -\bar{W }||. \end{aligned}$$

As consequence, the operator *F* has a distinct fixed point, therefore the results of the proposed fractional system (11) is exceptional.

Ulam–Hyers stability is a mathematical concept used to assess the behavior of a system. This stability notion is particularly relevant when dealing with fractional-order models because these equations can describe complex systems where memory effects and long-range interactions play a crucial role. In study of a fractional system, one of the main challenges is that exact analytical solutions are often difficult or even impossible to obtain. Instead, researchers commonly seek approximate or close-to-perfect solutions to gain insights into the system’s behavior. Ulam–Hyers stability, along with the extended Ulam–Hyers–Rassias stability definition, provides a useful framework to study the stability of approximate solutions of fractional system. It helps researchers understand the robustness of these solutions under small perturbations, which is crucial for evaluating the reliability of the model’s predictions. By employing Ulam–Hyers stability in the context of a system described by fractional differential equations, researchers can ascertain whether the approximate solutions obtained are reliable and meaningful. This stability analysis contributes to a deeper understanding of the system’s dynamics and aids in developing accurate and reliable mathematical models, which, in turn, are valuable for decision-making in various fields, such as epidemiology for infectious disease control and management.

## Ulam–Hyers stability

In this section, we will establish stability results for our recommended model of chikungunya introduced by Ulam^28^ and Hyers^29^. Numerous researchers used the Ulam–Hyers stability concept in various fields of science and engineering^30–32^. Here are some fundamental definitions and notions:

Consider a controller $N : {\mathcal {Z}} \rightarrow {\mathcal {Z}}$ in the following way

25 $$\begin{aligned} NW =W \ \ for \ \ W \in {\mathcal {Z}} \end{aligned}$$

### Definition 5.1

Previously (25) is Ulam–Hyers stable (UHS) kind if for every scenario $W \in Z$ and $\epsilon > 0$, we exhibit:

26 $$\begin{aligned} ||W -NW ||\le \epsilon \ \ \ for \ \ \ k \in [0,\upsilon ]. \end{aligned}$$

The situation (25) has a distinctive answer $\bar{W }$ in which $B_{q} > 0$ and the requirements listed below are met

27 $$\begin{aligned} ||\bar{W }-W ||\le B_{q} \epsilon , \ \ \ k \in [0,\upsilon ]. \end{aligned}$$

### Definition 5.2

The aforementioned (25) is a generalised UHS, thus we get the subsequent result for all possibilities $\bar{W }$ of (27) and any additional approach $\bar{W }$ of (25)

28 $$\begin{aligned} ||\bar{W }-W ||\le Y (\epsilon ), \end{aligned}$$

while $Y \ \in \ B(R, R)$ and the zero frame is zero.

### Remark 5.1

If the subsequent conditions are satisfied, the solution $\bar{W } \in Z$ accomplishes (27)

1. $$|\upsilon (k)| \le \epsilon , \ \ \forall \ \ k \in [0,\upsilon ]$$ where $$\upsilon \in B ([0,\upsilon ]; R)$$
2. ${\mathcal {G}}\bar{W }(F)=\bar{W }+\upsilon (F), \ \forall \ \ k \in [0,\upsilon ]$ Considering a minor disruption, the framework (11) may be expressed in the following manner
  29 $$\begin{aligned} \left\{ \begin{array}{rcl} ^{b}D_{0^{+}}^{\alpha }{} W (k)=&{} Q (k,W (k))+\upsilon (k),\\ W (0)=W _{0}. \end{array}\right. \end{aligned}$$

### Lemma 5.1

The given technique (29) meets the subsequent requirement

30 $$\begin{aligned} |W (k)-FW (k)|\le a \epsilon , \ \ where \ \ a=\frac{\upsilon ^{\alpha }}{\Gamma (\alpha +1)}. \end{aligned}$$

Proof 5.1. Employing Lemma (2.1) and Remark (5.1), the preceding conclusion is simply achieved.

### Theorem 5.1

If the criterion $\frac{\upsilon ^{\alpha }L_{Q }}{\Gamma (\alpha +1)}<1$ is met, the approach to the framework (11) is UHS and modified UHS on Lemma (5.1).

Proof 5.2. For the needed evidence, we consider the system response $W \in Z$ (29) and $\bar{W } \in F$ as an exceptional result (11), thus we get

31 $$\begin{aligned} |W (k)-\bar{W }(k)|= & {} |W (k)-\bar{W }(k)| \nonumber \\\le & {} |W (k)-F\bar{W }(k)| \nonumber \\\le & {} |W (k)-F\bar{W }(k)|\nonumber \\\le & {} a\epsilon +\frac{\upsilon ^{\alpha }L_{Q }}{\Gamma (\alpha +1)}|W (k)-\bar{W }(k)|\nonumber \\\le & {} \frac{a\epsilon }{1-\frac{\upsilon ^{\alpha }L_{Q }}{\Gamma (\alpha +1)}}. \end{aligned}$$

As a consequence, the chikungunya virus infection framework (11) indicated before is UHS and generalised UHS.

### Definition 5.3

Suppose $\omega \in B[[0, \upsilon ], R]$, then the challenge (25) is Ulam–Hyers–Rassias stable (UHRS) if each of the subsequent solutions $W \in Z$ is Ulam–Hyers–Rassias stable (UHRS)

32 $$\begin{aligned} ||W -NW ||\le \omega (k)\epsilon , \ for \ k \in [0,\upsilon ] \ and \ \epsilon > 0. \end{aligned}$$

One can discover an innovative approach $\bar{W }$ of the framework (25) that meets the constraint $B_{q} > 0$ providing

33 $$\begin{aligned} ||\bar{W }-W ||\le B_{q}\omega (k)\epsilon , \ \forall \ k \in [0,\upsilon ]. \end{aligned}$$

### Definition 5.4

Assume that $$W $$ be any solution of (32) and $$\bar{W }$$ is the unique solution of (25) in a manner that

34 $$\begin{aligned} ||\bar{W }-W ||\le B_{q,\omega }\omega (k)\epsilon , \ \ \forall \ \ k \in [0,\upsilon ]. \end{aligned}$$

in which $\epsilon > 0$ and $\omega \in B[[0,\upsilon ],R]$ so that $B_{q,\omega }$. The aforementioned (25) is therefore generalised UHRS.

### Remark 5.2

If the subsequent conditions are met, then the outcome of $\bar{W } \in X$ fulfils (27)

- $$|\upsilon (k)|\le \epsilon \omega (k), \ \forall \ k\in [0,\upsilon ]$$, where $$\upsilon (k) \in B([0, \upsilon ]; R)$$
- $$ N \bar{W }(k)=\bar{W }+\upsilon (k), \ \forall \ k\in [0,\upsilon ].$$

### Lemma 5.2

The inequality stated underneath holds valid for the perturb system (5.1) provided by

35 $$\begin{aligned} |W (k)-FW (F)|\le a\omega (k)\epsilon , \ with \ \ {\mathfrak {a}}=\frac{\upsilon ^{\alpha }}{\Gamma (\alpha +1)}. \end{aligned}$$

**Proof**. The needed consequence is simply shown utilising Lemma (2.1) and Remark (5.2).

### Theorem 5.2

Whenever $\frac{\upsilon ^{\alpha }L_{Q }}{\Gamma (\alpha +1)}<1$, then the answer to framework (11) is UHRS and generalised UHRS on Lemma (5.2).

Proof. If we presume a solution $W \in X$ and pick the particular answer $\bar{W } \in X$ of the framework (11), we get the following result

36 $$\begin{aligned} |W (k)-\bar{W }(k)|= & {} |W (k)-\bar{W }(k)| \\\\\le & {} |W (k)-F\bar{W }(k)| \nonumber \\\le & {} |W (k)-F\bar{W }(k)|\\\\\le & {} a\omega (k)\epsilon +\frac{\upsilon ^{\alpha } L_{Q }}{\Gamma (\alpha +1)}|W (k)-\bar{W }(k)|\nonumber \\\le & {} \frac{a\omega (k)\epsilon }{1-\frac{\upsilon ^{\alpha } L_{Q }}{\Gamma (\alpha +1)}}. \end{aligned}$$

As a result, UHRS and expanded UHRS are the answers to (11).

## Solution of our fractional system

In this section, we will utilise the transformation of Laplace to create an efficient scheme for our fractional system (9) of chikungunya infection. The Adomian Decomposition Method is an efficient and trustworthy numerical scheme, providing a dependable and effective approach for solving fractional models. This method exhibits excellent convergence properties, enabling researchers to obtain reliable approximations even when dealing with complex fractional models.

The following steps are involved in this scheme:

37 $$\begin{aligned} \left\{ \begin{array}{rcl} \pounds [S_{h}(k)]&{}=&{}\frac{S_{h0}}{s}+\frac{1}{s^{\vartheta }}\pounds \bigg [ \mu _h^{\xi } N_h -\frac{\beta _1 b^{\xi } S_hI_v}{N_h}-p^{\xi }S_h-\mu _h^{\xi } S_h \bigg ],\\ \pounds [V_{h}(k)]&{}=&{}\frac{V_{h0}}{s}+\frac{1}{s^{\vartheta }}\pounds \bigg [ p^{\xi }S_h-\frac{\beta _2 b^{\xi } V_h I_v}{N_h}-\mu _h^{\xi } V_h \bigg ],\\ \pounds [I_h(k)]&{}=&{}\frac{I_{h0}}{s}+\frac{1}{s^{\vartheta }}\pounds \bigg [ \frac{\beta _1 b^{\xi } S_h I_v}{N_h}+ \frac{\beta _2 b^{\xi } V_h I_v}{N_h}-(\tau ^{\xi }+\mu _h^{\xi }+\gamma ^{\xi }) I_h \bigg ],\\ \pounds [R_h(k)]&{}=&{}\frac{I_{h0}}{s}+\frac{1}{s^{\vartheta }}\pounds \bigg [ \tau ^{\xi } I_h+\gamma ^{\xi } I_h-\mu _h^{\xi } R_h \bigg ],\\ \pounds [S_{v}(k)]&{}=&{}\frac{S_{v0}}{s}+\frac{1}{s^{\vartheta }}\pounds \bigg [ \mu _v^{\xi } N_v-\frac{\beta _3 b^{\xi } S_v I_h}{N_h}-\mu _v^{\xi } S_v \bigg ],\\ \pounds [I_{v}(k)]&{}=&{}\frac{I_{v0}}{s}+\frac{1}{s^{\vartheta }}\pounds \bigg [\frac{\beta _3 b^{\xi } S_v I_h}{N_h}-\mu _v^{\xi } I_v \bigg ]. \end{array}\right. \end{aligned}$$

having

38 $$\begin{aligned} \left\{ \begin{array}{rcl} S_{h}(k)&{}=&{}\sum _{m=0}^{\infty }S_{hm}(k),\\ V_{h}(k)&{}=&{}\sum _{m=0}^{\infty }V_{hm}(k),\\ I_h(k)&{}=&{}\sum _{m=0}^{\infty }I_{hm}(k),\\ R_h(k)&{}=&{}\sum _{m=0}^{\infty }R_{hm}(k),\\ S_{v}(k)&{}=&{}\sum _{m=0}^{\infty }S_{vm}(k),\\ I_v(k)&{}=&{}\sum _{m=0}^{\infty }I_{vm}(k). \end{array}\right. \end{aligned}$$

Here, Adomian polynomials are used to separate the nonlinear components of our framework, and we progress in the following manner

$$\begin{aligned}{} & {} S_{h}(k)I_v(k)=\sum _{m=0}^{\infty }A_{m}(k), \ with \ A_{m}(k)=\frac{1}{m!}\frac{d^{m}}{dz^{m}}\bigg [\sum _{n=0}^{m}z^{k}S_{hk}(k) \sum _{n=0}^{m}z^{k}I_{hk}(k)\bigg ]z=0,\\{} & {} V_{h}(k)I_v(k)=\sum _{m=0}^{\infty }B_{m}(k),~where~B_{m}(k)=\frac{1}{m!}\frac{d^{m}}{dz^{m}}\bigg [\sum _{n=0}^{m}z^{k}V_{hk}(k) \sum _{n=0}^{m}z^{k}I_{vk}(k)\bigg ]z=0, \end{aligned}$$

and

$$\begin{aligned} S_v(k)(I_h(k))=\sum _{m=0}^{\infty }C_{m}(k),~where~C_{m}(k)=\frac{1}{m!}\frac{d^{m}}{dz^{m}}\bigg [\sum _{n=0}^{m}z^{k}S_{vk}(k) \sum _{n=0}^{m}z^{k}(I_{hk}(k))\bigg ]z=0, \end{aligned}$$

then, we obtain

39 $$\begin{aligned}{} & {} \left\{ \begin{array}{rcl} \pounds \bigg [\sum _{m=0}^{\infty }S_{hm}(k)\bigg ]&{}=&{}\frac{S_{h0}}{s}+\frac{1}{s^{\xi }}\pounds \bigg [\mu _h^{\xi } N_h -\frac{\beta _{1} b^{\xi } }{N_h}\sum _{m=0}^{\infty }A_{m}(k)-p^{\xi } \sum _{m=0}^{\infty }S_{hm}(k)-\mu _h^{\xi } \sum _{m=0}^{\infty }S_{hm}(k) \bigg ],\\ \pounds \bigg [\sum _{m=0}^{\infty }V_{hm}(k)\bigg ]&{}=&{}\frac{V_{h0}}{s}+\frac{1}{s^{\xi }}\pounds \bigg [ p^{\xi } \sum _{m=0}^{\infty }S_{hm}(k)-\frac{\beta _{2} b^{\xi } }{N_h}\sum _{m=0}^{\infty }B_{m}(k)-\mu _h^{\xi } \sum _{m=0}^{\infty }V_{hm}(k) \bigg ],\\ \pounds \bigg [\sum _{m=0}^{\infty }I_{hm}(k)\bigg ]&{}=&{}\frac{I_{h0}}{s}+\frac{1}{s^{\xi }}\pounds \bigg [ \frac{\beta _{h} b^{\xi } }{N_h} \sum _{m=0}^{\infty }A_{m}(k) +\frac{\beta _{2} b^{\xi } }{N_h}\sum _{m=0}^{\infty }B_{m}(k)-(\mu _h^{\xi } +\tau ^{\xi }+\rho ^{\xi } ) \sum _{m=0}^{\infty }I_{hm}(k) \bigg ],\\ \pounds \bigg [\sum _{m=0}^{\infty }R_{hm}(k)\bigg ]&{}=&{}\frac{R_{h0}}{s}+\frac{1}{s^{\xi }}\pounds \bigg [ (\tau ^{\xi }+\gamma ^{\xi }) \sum _{m=0}^{\infty } I_{hm} - \mu _h^\xi \sum _{m=0}^{\infty }R_{hm}(k) \bigg ],\\ \pounds \bigg [\sum _{m=0}^{\infty }S_{vm}(k)\bigg ]&{}=&{}\frac{S_{v0}}{s}+\frac{1}{s^{\xi }}\pounds \bigg [ \mu _v^{\vartheta } N_v -\frac{\beta _v b^{\vartheta } }{N_h}\sum _{m=0}^{\infty }C_{m}(k) -\mu _v^{\vartheta } \sum _{m=0}^{\infty }S_{vm}(k) \bigg ],\\ \pounds \bigg [\sum _{m=0}^{\infty }I_{vm}(k)\bigg ]&{}=&{}\frac{I_{v0}}{s}+\frac{1}{s^{\xi }}\pounds \bigg [ \frac{\beta _v b^{\xi } }{N_h}\sum _{m=0}^{\infty }C_{m}(k) - \mu _v^{\xi } \sum _{m=0}^{\infty }I_{vm}(k) \bigg ]. \end{array}\right. \end{aligned}$$

40 $$\begin{aligned}{} & {} \left\{ \begin{array}{lll} \pounds [S_{h}(k)]&{}=&{}\frac{S_{h0}}{s},\\ \pounds [V_{h0}(k)]&{}=&{}\frac{V_{h0}}{s},\\ \pounds [I_{h0}(k)]&{}=&{}\frac{I_{h0}}{s},\\ \pounds [R_{h0}(k)]&{}=&{}\frac{R_{h0}}{s},\\ \pounds [S_{v0}(k)]&{}=&{}\frac{S_{v0}}{s},\\ \pounds [I_{v0}(k)]&{}=&{}\frac{I_{v0}}{s}. \end{array}\right. \end{aligned}$$

41 $$\begin{aligned}{} & {} \left\{ \begin{array}{rcl} \pounds [S_{h1}(k)]&{}=&{}\frac{1}{s^{\xi }}\pounds \bigg [\mu _h^{\xi } N_h -\frac{\beta _{1} b^{\xi } }{N_h}A_0(k)-p^{\xi } S_{h0}(k)-\mu _h^{\xi } S_{h0}(k)\bigg ],\\ \pounds [V_{h1}(k)]&{}=&{}\frac{1}{s^{\xi }}\pounds \bigg [ p^{\xi } S_{h0}(k)-\frac{\beta _{2} b^{\xi } }{N_h} B_0(k)-\mu _h^{\xi } V_{h0}(k) \bigg ],\\ \pounds [I_{h1}(k)]&{}=&{}\frac{1}{s^{\xi }}\pounds \bigg [\frac{\beta _{1} b^{\xi } }{N_h}A_0(k)+\frac{\beta _{2} b^{\xi } }{N_h}B_0(k)-(\mu _h^{\xi } +\gamma ^{\xi }+\tau ^{\xi } ) I_{h0}(k) \bigg ],\\ \pounds [R_{h1}(k)]&{}=&{}\frac{1}{s^{\xi }}\pounds \bigg [ \gamma ^{\xi } I_{h0}(k)+\tau ^{\xi } I_{h0}(k)-\mu _h^{\xi } R_{h0}(k) \bigg ],\\ \pounds [S_{v1}(k)]&{}=&{}\frac{1}{s^{\xi }}\pounds \bigg [\mu _v^{\xi } N_v -\frac{\beta _v b^{\xi } }{N_h}C_0(k) -\mu _v^{\xi } S_{v0}(k) \bigg ],\\ \pounds [I_{v1}(k)]&{}=&{}\frac{1}{s^{\xi }}\pounds \bigg [ \frac{\beta _3 b^{\xi } }{N_h}C_0(k) - \mu _v^{\xi } I_{v0}(k) \bigg ],\\ \end{array}\right. \end{aligned}$$

and

42 $$\begin{aligned} \left\{ \begin{array}{rcl} \pounds [S_{h2}(k)]&{}=&{}\frac{1}{s^{\xi }}\pounds \bigg [\mu _h^{\xi } N_h -\frac{\beta _{1} b^{\xi } }{N_h}A_1(k)-p^{\xi } S_{h1}(k)-\mu _h^{\xi } S_{h1}(k)\bigg ],\\ \pounds [V_{h2}(k)]&{}=&{}\frac{1}{s^{\xi }}\pounds \bigg [ p^{\xi } S_{h1}(k)-\frac{\beta _{2} b^{\xi } }{N_h} B_1(k)-\mu _h^{\xi } V_{h1}(k) \bigg ],\\ \pounds [I_{h2}(k)]&{}=&{}\frac{1}{s^{\xi }}\pounds \bigg [\frac{\beta _{1} b^{\xi } }{N_h}A_1(k)+\frac{\beta _{2} b^{\xi } }{N_h}B_1(k)-(\mu _h^{\xi } +\gamma ^{\xi }+\tau ^{\xi } ) I_{h1}(k) \bigg ],\\ \pounds [R_{h2}(k)]&{}=&{}\frac{1}{s^{\xi }}\pounds \bigg [ \gamma ^{\xi } I_{h1}(k)+\tau ^{\xi } I_{h1}(k)-\mu _h^{\xi } R_{h1}(k) \bigg ],\\ \pounds [S_{v2}(k)]&{}=&{}\frac{1}{s^{\xi }}\pounds \bigg [\mu _v^{\xi } N_v -\frac{\beta _v b^{\xi } }{N_h}C_1(k) -\mu _v^{\xi } S_{v1}(k) \bigg ],\\ \pounds [I_{v2}(k)]&{}=&{}\frac{1}{s^{\xi }}\pounds \bigg [ \frac{\beta _3 b^{\xi } }{N_h}C_1(k) - \mu _v^{\xi } I_{v1}(k) \bigg ],\\ \end{array}\right. \end{aligned}$$

so on, we have

43 $$\begin{aligned} \left\{ \begin{array}{rcl} \pounds [S_{h(m+1)}(k)]&{}=&{}\frac{1}{s^{\xi }}\pounds \bigg [\mu _h^{\xi } N_h -\frac{\beta _{1} b^{\xi } }{N_h}A_m(k)-p^{\xi } S_{hm}(k)-\mu _h^{\xi } S_{hm}(k)\bigg ],\\ \pounds [V_{h(m+1)}(k)]&{}=&{}\frac{1}{s^{\xi }}\pounds \bigg [ p^{\xi } S_{h0}(k)-\frac{\beta _{2} b^{\xi } }{N_h} B_m(k)-\mu _h^{\xi } V_{hm}(k) \bigg ],\\ \pounds [I_{h(m+1)}(k)]&{}=&{}\frac{1}{s^{\xi }}\pounds \bigg [\frac{\beta _{1} b^{\xi } }{N_h}A_m(k)+\frac{\beta _{2} b^{\xi } }{N_h}B_m(k)-(\mu _h^{\xi } +\gamma ^{\xi }+\tau ^{\xi } ) I_{hm}(k) \bigg ],\\ \pounds [R_{h(m+1)}(k)]&{}=&{}\frac{1}{s^{\xi }}\pounds \bigg [ \gamma ^{\xi } I_{hm}(k)+\tau ^{\xi } I_{hm}(k)-\mu _h^{\xi } R_{hm}(k) \bigg ],\\ \pounds [S_{v(m+1)}(k)]&{}=&{}\frac{1}{s^{\xi }}\pounds \bigg [\mu _v^{\xi } N_v -\frac{\beta _v b^{\xi } }{N_h}C_m(k) -\mu _v^{\xi } S_{vm}(k) \bigg ],\\ \pounds [I_{v(m+1)}(k)]&{}=&{}\frac{1}{s^{\xi }}\pounds \bigg [ \frac{\beta _3 b^{\xi } }{N_h}C_m(k) - \mu _v^{\xi } I_{vm}(k) \bigg ],\\ \end{array}\right. \end{aligned}$$

with the initial conditions

44 $$\begin{aligned} \left\{ \begin{array}{rcl} S_{h0}(k)&{}=&{}S_{h0}\\ V_{h0}(k)&{}=&{}V_{h0}\\ I_{h0}(k)&{}=&{} I_{h0}\\ R_{h0}(k)&{}=&{} R_{h0}\\ S_{v0}(k)&{}=&{}S_{v0}\\ I_{v0}(k)&{}=&{}I_{v0} \end{array}\right. \end{aligned}$$

To further simplify things, we perform an additional process

45 $$\begin{aligned} \left\{ \begin{array}{rcl} S_{h1}(k)&{}=&{} \pounds ^{-1}\bigg [ \frac{1}{s^{\xi }}\pounds \bigg [\mu _h^{\xi } N_h -\frac{\beta _{1} b^{\xi } }{N_h}A_0(k)-p^{\xi } S_{h0}(k)-\mu _h^{\xi } S_{h0}(k)\bigg ] \bigg ],\\ V_{h1}(k)&{}=&{} \pounds ^{-1}\bigg [\frac{1}{s^{\xi }}\pounds \bigg [ p^{\xi } S_{h0}(k)-\frac{\beta _{2} b^{\xi } }{N_h} B_0(k)-\mu _h^{\xi } V_{h0}(k) \bigg ] \bigg ],\\ I_{h1}(k) &{}=&{} \pounds ^{-1}\bigg [ \frac{1}{s^{\xi }}\pounds \bigg [\frac{\beta _{1} b^{\xi } }{N_h}A_0(k)+\frac{\beta _{2} b^{\xi } }{N_h}B_0(k)-(\mu _h^{\xi } +\gamma ^{\xi } +\tau ^\xi ) I_{h0}(k) \bigg ] \bigg ],\\ R_{h1}(k)&{}=&{} \pounds ^{-1}\bigg [\frac{1}{s^{\xi }}\pounds \bigg [ ( \gamma ^{\xi } +\tau ^\xi ) I_{h0}(k)-(\mu _h^{\xi }) R_{h0}(k) \bigg ] \bigg ],\\ S_{v1}(k)&{}=&{} \pounds ^{-1}\bigg [ \frac{1}{s^{\xi }}\pounds \bigg [\mu _v^{\xi } N_v -\frac{\beta _3 b^{\xi } }{N_h}C_0(k) -\mu _v^{\xi } S_{v0}(k) \bigg ] \bigg ],\\ I_{v1}(k)&{}=&{} \pounds ^{-1}\bigg [ \frac{1}{s^{\xi }}\pounds \bigg [ \frac{\beta _3 b^{\xi } }{N_h}C_0(k) - \mu _v^{\xi } I_{v0}(k) \bigg ] \bigg ], \end{array}\right. \end{aligned}$$

and

46 $$\begin{aligned} \left\{ \begin{array}{rcl} S_{h2}(k)&{}=&{} \pounds ^{-1}\bigg [ \frac{1}{s^{\xi }}\pounds \bigg [\mu _h^{\xi } N_h -\frac{\beta _{1} b^{\xi } }{N_h}A_1(k)-p^{\xi } S_{h1}(k)-\mu _h^{\xi } S_{h1}(k)\bigg ] \bigg ],\\ V_{h2}(k)&{}=&{} \pounds ^{-1}\bigg [\frac{1}{s^{\xi }}\pounds \bigg [ p^{\xi } S_{h1}(k)-\frac{\beta _{2} b^{\xi } }{N_h} B_1(k)-\mu _h^{\xi } V_{h1}(k) \bigg ] \bigg ],\\ I_{h2}(k) &{}=&{} \pounds ^{-1}\bigg [ \frac{1}{s^{\xi }}\pounds \bigg [\frac{\beta _{1} b^{\xi } }{N_h}A_1(k)+\frac{\beta _{2} b^{\xi } }{N_h}B_1(k)-(\mu _h^{\xi } +\gamma ^{\xi } +\tau ^\xi ) I_{h1}(k) \bigg ] \bigg ],\\ R_{h2}(k)&{}=&{} \pounds ^{-1}\bigg [\frac{1}{s^{\xi }}\pounds \bigg [ ( \gamma ^{\xi } +\tau ^\xi ) I_{h1}(k)-(\mu _h^{\xi }) R_{h1}(k) \bigg ] \bigg ],\\ S_{v2}(k)&{}=&{} \pounds ^{-1}\bigg [ \frac{1}{s^{\xi }}\pounds \bigg [\mu _v^{\xi } N_v -\frac{\beta _3 b^{\xi } }{N_h}C_1(k) -\mu _v^{\xi } S_{v1}(k) \bigg ] \bigg ],\\ I_{v2}(k)&{}=&{} \pounds ^{-1}\bigg [ \frac{1}{s^{\xi }}\pounds \bigg [ \frac{\beta _3 b^{\xi } }{N_h}C_1(k) - \mu _v^{\xi } I_{v1}(k) \bigg ] \bigg ], \end{array}\right. \end{aligned}$$

so on, we have

47 $$\begin{aligned} \left\{ \begin{array}{rcl} S_{h(m+1)}(k)&{}=&{} \pounds ^{-1}\bigg [ \frac{1}{s^{\xi }}\pounds \bigg [\mu _h^{\xi } N_h -\frac{\beta _{1} b^{\xi } }{N_h}A_m(k)-p^{\xi } S_{hm}(k)-\mu _h^{\xi } S_{hm}(k)\bigg ] \bigg ],\\ V_{h(m+1)}(k)&{}=&{} \pounds ^{-1}\bigg [\frac{1}{s^{\xi }}\pounds \bigg [ p^{\xi } S_{hm}(k)-\frac{\beta _{2} b^{\xi } }{N_h} B_m(k)-\mu _h^{\xi } V_{hm}(k) \bigg ] \bigg ],\\ I_{h(m+1)}(k) &{}=&{} \pounds ^{-1}\bigg [ \frac{1}{s^{\xi }}\pounds \bigg [\frac{\beta _{1} b^{\xi } }{N_h}A_m(k)+\frac{\beta _{2} b^{\xi } }{N_h}B_m(k)-(\mu _h^{\xi } +\gamma ^{\xi } +\tau ^\xi ) I_{hm}(k) \bigg ] \bigg ],\\ R_{h(m+1)}(k)&{}=&{} \pounds ^{-1}\bigg [\frac{1}{s^{\xi }}\pounds \bigg [ ( \gamma ^{\xi } +\tau ^\xi ) I_{hm}(k)-(\mu _h^{\xi }) R_{hm}(k) \bigg ] \bigg ],\\ S_{v(m+1)}(k)&{}=&{} \pounds ^{-1}\bigg [ \frac{1}{s^{\xi }}\pounds \bigg [\mu _v^{\xi } N_v -\frac{\beta _3 b^{\xi } }{N_h}C_m(k) -\mu _v^{\xi } S_{vm}(k) \bigg ] \bigg ],\\ I_{v(m+1)}(k)&{}=&{} \pounds ^{-1}\bigg [ \frac{1}{s^{\xi }}\pounds \bigg [ \frac{\beta _3 b^{\xi } }{N_h}C_m(k) - \mu _v^{\xi } I_{vm}(k) \bigg ] \bigg ]. \end{array}\right. \end{aligned}$$

Ultimately, we obtain the following solution in series form

48 $$\begin{aligned} \left\{ \begin{array}{rcl} S_{h}(k)&{}=&{}S_{h0}(k)+S_{h1}(k)+S_{h2}(k)+S_{h3}(k)+\dots ,\\ \\ V_{h}(k)&{}=&{}V_{h0}(k)+V_{h1}(k)+V_{h2}(k)+V_{h3}(k)+\dots ,\\ \\ I_h(k)&{}=&{}I_{h0}(k)+I_{h1}(k)+I_{h2}(k)+I_{h3}(k)+\dots ,\\ \\ R_h(k)&{}=&{}R_{h0}(k)+R_{h1}(k)+R_{h2}(k)+R_{h3}(k)+\dots ,\\ \\ S_v(k)&{}=&{}S_{v0}(k)+S_{v1}(k)+S_{v2}(k)+S_{v3}(k)+\dots ,\\ \\ I_v(k)&{}=&{}I_{v0}(k)+I_{v1}(k)+I_{v2}(k)+I_{v3}(k)+\dots ~. \end{array}\right. \end{aligned}$$

**Figure 2:**
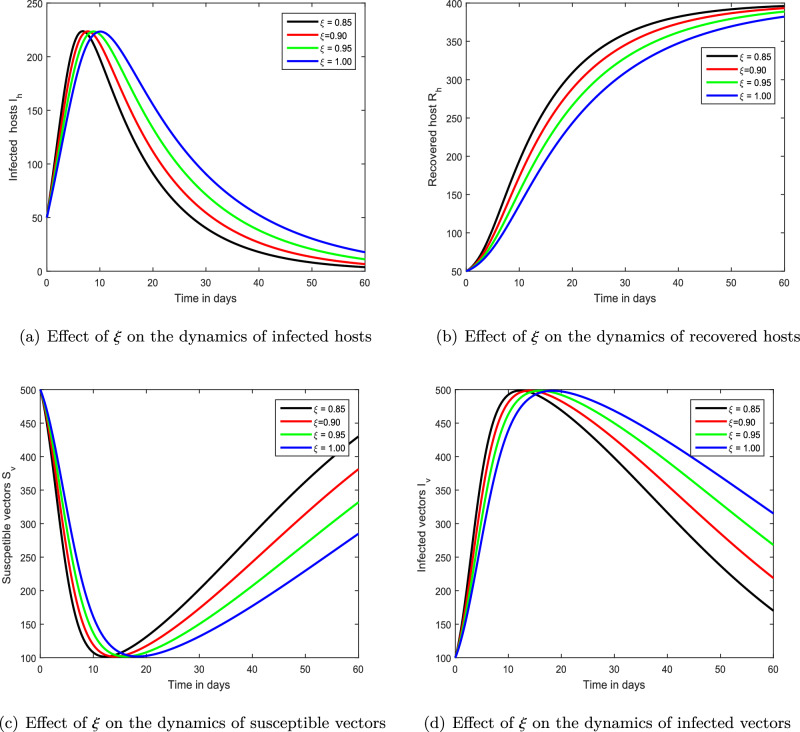
Demonstration of the dynamical behaviour of our chikungunya framework (9) with varying memory index $\xi$ values, i.e., $\xi =0.85,0.90,0.95,1.00$.

**Figure 3:**
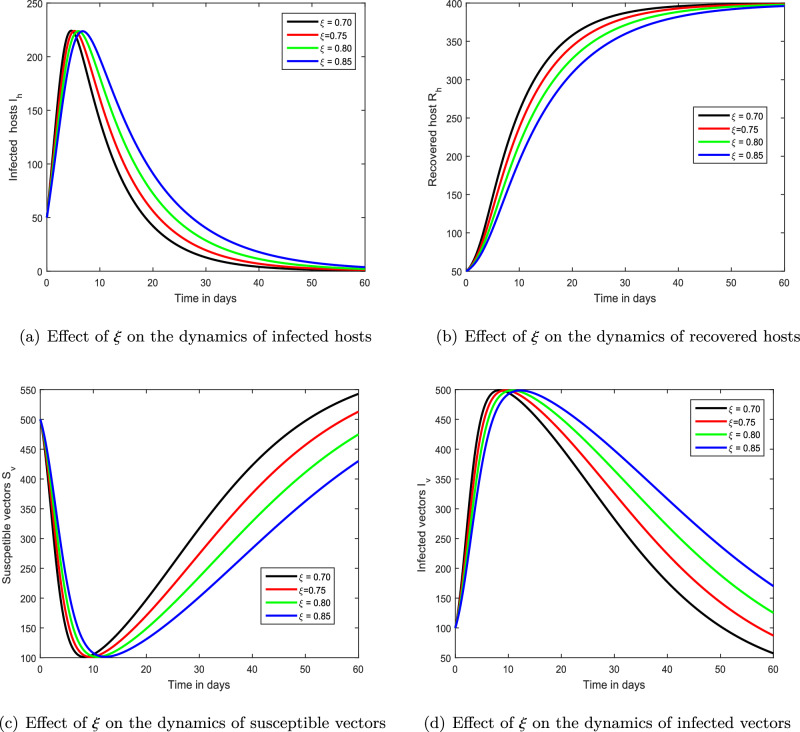
Demonstration of the dynamical response of our chikungunya framework (9) with various fractal order $\xi$ values, i.e., $\xi =0.70,0.75,0.80,0.85$.

**Figure 4:**
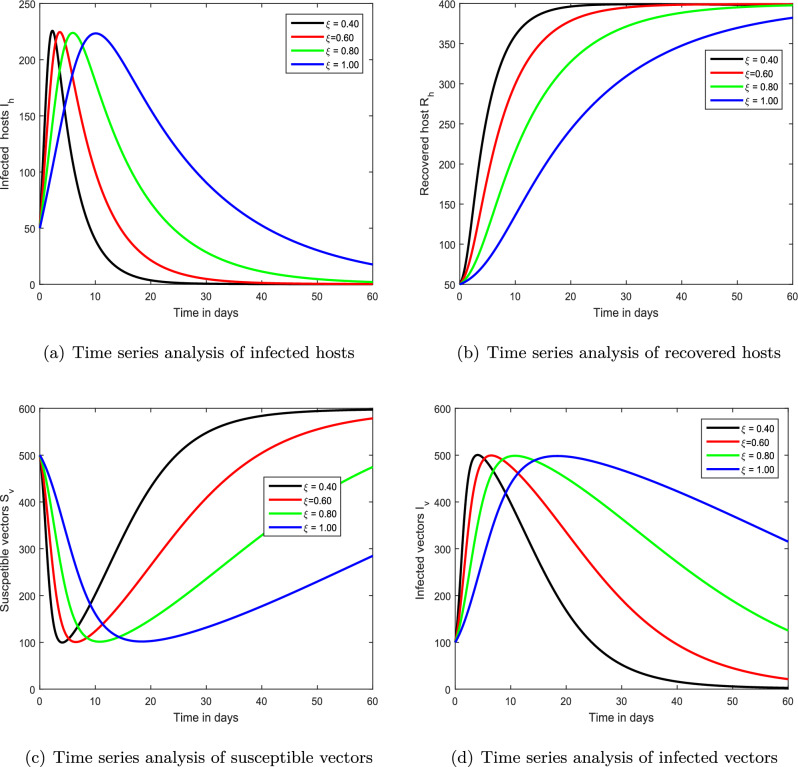
Demonstration of the dynamical behaviour of our chikungunya framework (9) with varying memory index $\xi$ values, i.e., $\xi =0.4,0.6,0.8,1.0$.

**Figure 5:**
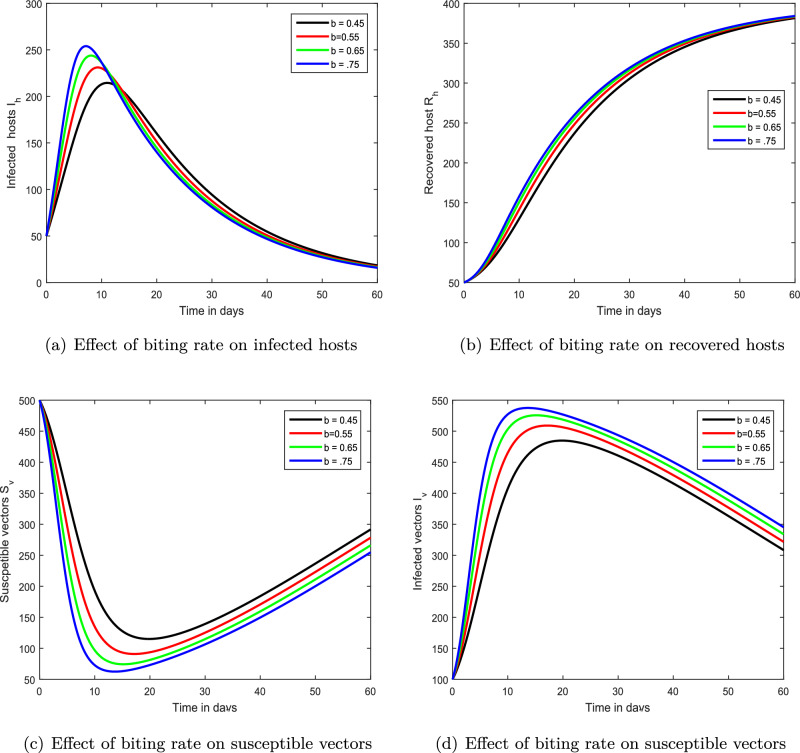
Demonstration of the dynamical behaviour of our chikungunya framework (9) with varying vector biting rate values, i.e., $b=0.45,0.55,0.65,0.75$.

**Figure 6:**
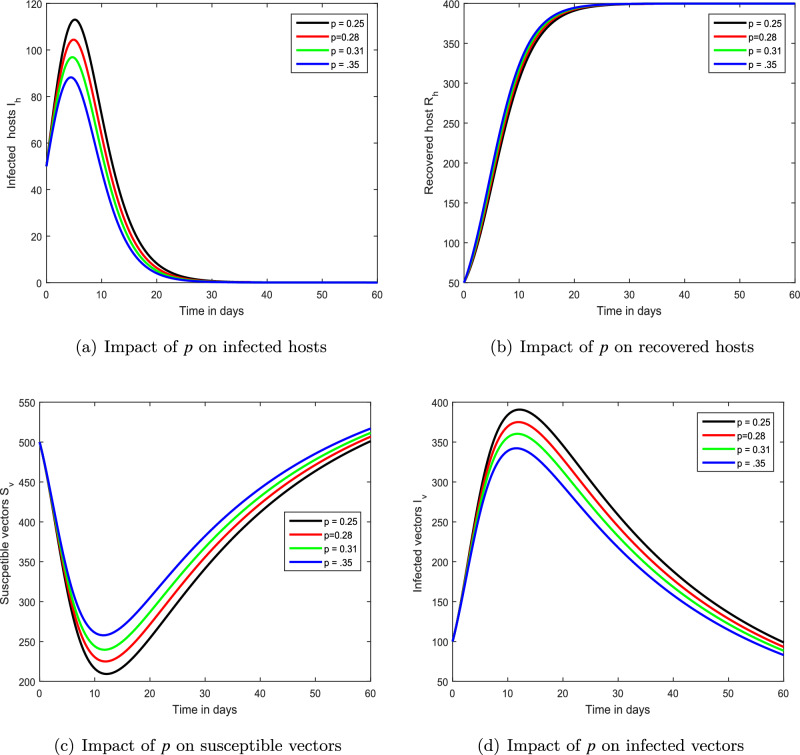
Demonstration of the dynamical behaviour of our chikungunya framework (9) with various vaccination levels *p*, i.e., $p=0.25,0.28,0.31,0.35$.

**Figure 7:**
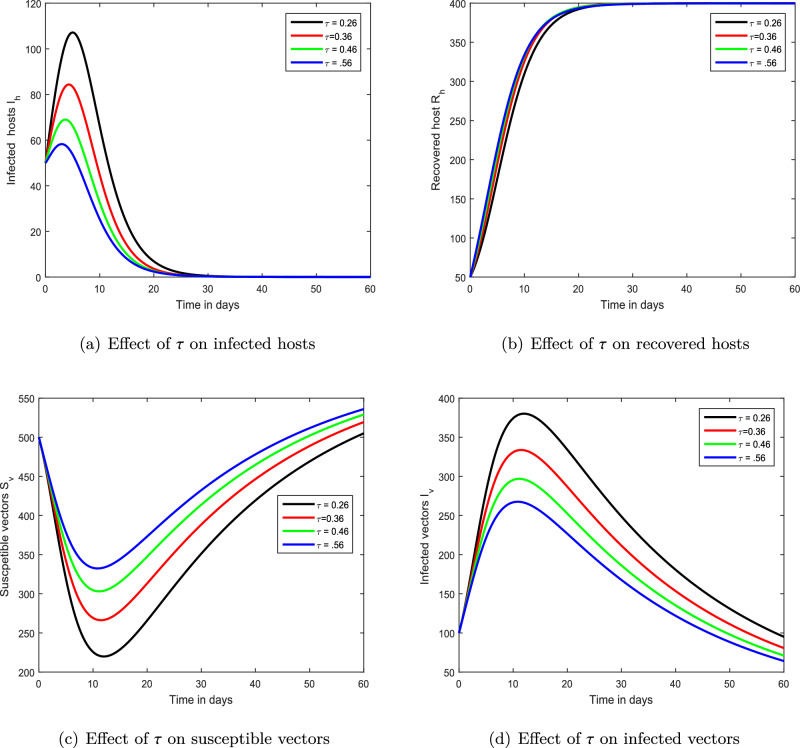
Demonstration of the dynamical behaviour of our chikungunya framework (9) using different treatment rate $\tau$ values, i.e., $\tau =0.26,0.36,0.46,0.56$.

The above scheme is described earlier is used to calculate the numerical findings of our fractional system (9) of chikungunya infection. We estimated the system’s input parameter contents for numerical objectives. Through numerical simulations, we want to illustrate the influence of the input component on the dynamics of chikungunya. In light of our findings, we will propose efficient prevention strategies to lower the prevalence of chikungunya in the community. We provided a time series study of the suggested mechanism of chikungunya infection in these computations.

We exhibited the dynamical performance of the suggested fractional framework for chikungunya infection with the modification of the index of memory in the first interference, shown in Figs. 2, 3 and 4. In Fig. 2, we assumed $\xi$ to be 0.85, 0.90, 0.95 and 1.00 while the parameter $\xi$ is considered to be 0.70, 0.75, 0.80, 0.85 and 0.4, 0.6, 0.8, 1.00 in Figs. 3 and 4, respectively. According to our findings, the infection level of both species reduces as the memory index lowers. As a result, we propose that controlling the memory index could regulate this viral infection and propose it to authorities for the avoidance of infections in the community. In the second simulation illustrated in Fig. 5, we emphasized the solution paths of the chikungunya virus infection model with biting rate variation. We discovered that when the bite rate boosted so did the degree of illness. As a result, this input component is crucial.

We demonstrated the influence of vaccination on the propagation dynamics of chikungunya viral infection in the third simulation depicted in Fig. 6. We discovered that immunization is a key element in reducing chikungunya infection in the community. The vaccine parameters are advised to health officials for infection control based on these findings. The impact of treatment has been visualized using numerical simulations in the final simulation shown in Fig. 7. We proposed that the index of memory, immunization, and treatment be utilized as control criteria for chikungunya viral infection avoidance. In our future work, the proposed model will be examined and validated using real-world data on the availability of data, enabling us to to predict the future course of the epidemic.

## Concluding remarks

In this study, we developed a mathematical framework for the transmission of chikungunya infection with vaccination and therapy. The suggested chikungunya model is constructed in a fractional framework to demonstrate the influence of memory on the dynamics of chikungunya. We used the basic principles of fractional calculus to analyze our mathematical framework. In our study, we focused on qualitative analysis and the dynamical behavior of chikungunya viral infection. The uniqueness and existence of the solution of the provided chikungunya model are investigated using the fixed-point theorem within the context of Banach’s and Schaefer’s. Through these, we obtained the Ulam–Hyers stability criteria for our chikungunya viral infection system. The influence of various factors on the dynamics of chikungunya virus infection is investigated by employing the Laplace Adomian reduction methodology to demonstrate the impact of many parameters on the dynamics of this viral infection. Numerical simulations, in particular, are employed to illustrate the impacts of fractional-order, immunization, and treatment. We have shown that the bite rate is an important metric that can render a more challenging controller. The biting rate of mosquitos is expected to be harmful, whereas vaccines and treatment are promising characteristics for infection management. It has been proposed that reducing mosquito bite rates can reduce the severity of chikungunya virus illness. We demonstrated the role of memory in the dynamics of chikungunya infection and propose that it might be employed as a control measure for the prevention of infection. In addition to this, we hypothesized that chikungunya within society could be managed by reducing bite rates and enhancing vaccine and treatment.

## Data Availability

The data sets used and analysed during the current study is available from the corresponding author on reasonable request.
